# Glycated Albumin‐Guided Therapy: Biomarker Utility or Earlier Treatment Intensification?

**DOI:** 10.1111/1753-0407.70274

**Published:** 2026-09-28

**Authors:** Muhammet Fatih Şahin

**Affiliations:** ^1^ Department of Internal Medicine Kestel State Hospital Kestel Bursa Türkiye

Dear Editor,

We read with great interest the randomized controlled trial by Ren et al. evaluating glycated albumin (GA)‐guided treatment in patients with newly diagnosed type 2 diabetes mellitus (T2DM) [1]. The authors should be commended for conducting a multicenter randomized trial addressing an important question: whether a biomarker reflecting shorter‐term glycemic exposure can facilitate earlier therapeutic decisions. Nevertheless, several aspects warrant consideration when interpreting the clinical utility of the proposed GA‐guided strategy.

First, the primary endpoint was unequivocally neutral. The proportion of participants achieving HbA1c <7% at 12 weeks was virtually identical between the GA‐guided and standard‐treatment groups (64.3% vs. 64.4%; *p* = 0.99). In contrast, much of the suggested benefit derives from secondary outcomes, including changes in HbA1c, HOMA‐β, HOMA‐IR, anthropometric measures, and the more stringent HbA1c target. Given the number of secondary comparisons, clarification regarding adjustment for multiplicity would be valuable, as nominally significant secondary findings should be interpreted cautiously when the prespecified primary endpoint is negative.

Second, the reported advantage for achieving HbA1c <6.5% deserves particular attention. In the full analysis set, the difference did not reach conventional statistical significance (35.7% vs. 23.8%; *p* = 0.065), whereas significance emerged in the per‐protocol analysis (40.5% vs. 25.3%; *p* = 0.034). Because randomization primarily supports inference according to the intention‐to‐treat principle, emphasizing the per‐protocol result may overstate the robustness of this secondary finding.

More importantly, the trial raises a mechanistic question: does it demonstrate the clinical value of GA itself, or the benefit of earlier treatment intensification triggered by GA? Combination therapy increased from 23.5% to 63.7% in the GA‐guided group, while remaining approximately 18%–19% in the standard‐treatment group [1]. Thus, differences in HbA1c reduction, body weight, HOMA‐IR, and HOMA‐β may largely reflect greater treatment intensity rather than an independent advantage conferred by GA measurement. Previous trials such as VERIFY have already demonstrated potential benefits of early combination therapy in newly diagnosed T2DM [2]. A design comparing GA‐guided intensification with protocolized early intensification based on readily available glycemic measures could better isolate the incremental clinical value of GA.

Finally, improvements in HOMA‐β and HOMA‐IR over only 12 weeks should not necessarily be interpreted as evidence of durable β‐cell recovery or improved insulin sensitivity. These indices may change rapidly following amelioration of glucotoxicity, weight reduction, and treatment intensification.

Ren et al. provide valuable evidence regarding the feasibility of GA‐guided management. However, the present findings may more strongly support timely therapeutic intensification than establish GA itself as a clinically superior decision‐making biomarker. Longer‐term trials designed to separate the effect of the biomarker from that of treatment intensification are warranted.

## Author Contributions

**Muhammet Fatih Şahin** contributed to the conceptualization, literature review, interpretation of the relevant evidence, drafting of the manuscript, and critical revision of the manuscript. The author approved the final version and agrees to be accountable for all aspects of the work.

## Funding

The author has nothing to report.

## Conflicts of Interest

The author declare no conflicts of interest.

## Data Availability

Data sharing not applicable to this article as no datasets were generated or analysed during the current study.
